# Exposure of anti-infective drugs and the dynamic changes of the gut microbiota during gastrointestinal mucositis in autologous stem cell transplant patients: a pilot study

**DOI:** 10.1007/s00277-023-05091-y

**Published:** 2023-01-17

**Authors:** Anne-Grete Märtson, Ana Rita da Silva Ferreira, Anette Veringa, Lei Liu, Hannah R. Wardill, Lenneke A. T. Junier, Tjip S. van der Werf, Hermie J. M. Harmsen, Marieke G. G. Sturkenboom, Lambert F. Span, Wim J. E. Tissing, Jan-Willem C. Alffenaar

**Affiliations:** 1 Department of Clinical Pharmacy and Pharmacology, University Medical Center Groningen, University of Groningen, Groningen, The Netherlands; 2 Department of Medical Microbiology and Infection Prevention, University Medical Center Groningen, University of Groningen, Groningen, The Netherlands; 3 Adelaide Medical School, The University of Adelaide, Adelaide, Australia; 4 Precision Medicine (Cancer), South Australian Health and Medical Research Institute, Adelaide, Australia; 5 Department of Pulmonary Diseases and Tuberculosis, University Medical Center Groningen, University of Groningen, Groningen, The Netherlands; 6 Department of Internal Medicine, University Medical Center Groningen, University of Groningen, Groningen, The Netherlands; 7 Department of Hematology, University Medical Center Groningen, University of Groningen, Groningen, The Netherlands; 8 Department of Pediatrics (Oncology and Hematology), University of Groningen, University Medical Center Groningen, Groningen, The Netherlands; 9 Princess Maxima Centre for Pediatric Oncology, Utrecht, The Netherlands; 10 School of Pharmacy, Faculty of Medicine and Health, University of Sydney, Sydney, NSW Australia; 11 Westmead Hospital, Sydney, NSW Australia; 12 Marie Bashir Institute of Infectious Diseases and Biosecurity, University of Sydney, Sydney, NSW Australia

**Keywords:** Gastrointestinal mucositis, Autologous transplant, Stem cell transplantation, Microbiota, Antibiotic exposure

## Abstract

Gastrointestinal mucositis could potentially compromise drug absorption due to functional loss of mucosa and other pathophysiological changes in the gastrointestinal microenvironment. Little is known about this effect on commonly used anti-infectives. This study aimed to explore the association between different stages of gastrointestinal mucositis, drug exposure, and gut microbiota. A prospective, observational pilot study was performed in HSCT patients aged ≥ 18 years receiving anti-infectives orally. Left-over blood samples and fecal swabs were collected from routine clinical care until 14 days after HSCT to analyze drug and citrulline concentrations and to determine the composition of the gut microbiota. 21 patients with a median age of 58 (interquartile range 54–64) years were included with 252 citrulline, 155 ciprofloxacin, 139 fluconazole, and 76 acyclovir concentrations and 48 fecal swabs obtained. Severe gastrointestinal mucositis was observed in all patients. Due to limited data correlation analysis was not done for valacyclovir and fluconazole, however we did observe a weak correlation between ciprofloxacin and citrulline concentrations. This could suggest that underexposure of ciprofloxacin can occur during severe mucositis. A follow-up study using frequent sampling rather than the use of left-over would be required to investigate the relationship between gastrointestinal mucositis, drug exposure, and gut microbiome.

**Supplementary Information:**

The online version contains supplementary material available at 10.1007/s00277-023-05091-y.

## Introduction

In autologous hematopoietic stem cell transplantation (HSCT) recipients, gastrointestinal mucositis is a severe complication of certain cytotoxic chemotherapeutic agents. This condition is characterized by severe ulcerations and diffuse inflammation throughout the gastrointestinal tract [1]. The agents that are most often associated with mucositis are alkylating agents (e.g., melphalan and cyclophosphamide), anthracyclines (e.g., doxorubicin), antimetabolites (e.g., 5-fluorouracil), antitumor agents (e.g., bleomycin), taxanes, and vinca alkaloids [2]. Clinically, this complication manifests as oral mucositis with ulcers and pain, diarrhea, and abdominal pain.

Due to the gastrointestinal mucositis and level of immunosuppression, HSCT recipients are highly susceptible to blood stream infections [1], which arise from translocation of pathogens from the gut and exogenous contaminants (e.g., catheters) [3]. Prophylaxis is therefore routinely used to prevent infections [4]. As gastrointestinal mucositis affects the integrity of the mucosa, a decrease of the absorptive capacity and increase of permeability occur [5], coupled with changes in the microbial ecosystem of the gastrointestinal tract [6, 7]. The selection of oral or nous drug administration in people with gastrointestinal mucositis may have clinical consequences.

It is poorly understood how gastrointestinal mucositis may affect anti-infective drug absorption because of the variable and semiquantitative assessment of mucositis in daily practice as this has not been studied [8]. We do, however, hypothesize that intestinal barrier disruption during gastrointestinal mucositis could lead to an impaired intestinal barrier function, resulting in the alteration of the efficacy of anti-infectives commonly administrated in cancer patients. Plasma citrulline—a biomarker of enterocyte mass—can be used to objectively monitor and quantify gastrointestinal mucositis severity [9, 10]. Citrulline is a nonprotein amino acid produced in the enterocytes by glutamine, which has been increasingly shown to correlate with the intestinal villus length and with radiation-induced and chemotherapy-induced (e.g., methotrexate and melphalan) mucositis in mouse and rat models [11–13]. In HSCT recipients, low citrulline concentrations have been related to severe mucosal barrier injury and showed a strong correlation with the daily gut score [14–16].

Moreover, given the lack of consensus and insight into how gastrointestinal mucositis and secondary microbiome disruption influence the absorption and bioavailability of antimicrobial drugs [17, 18], the aim of our study was to investigate the relationship between plasma concentrations of ciprofloxacin, fluconazole, and valacyclovir, with gastrointestinal mucositis severity (defined by citrulline) and gut microbiome composition.

## Materials and methods

### Study design

A prospective, observational pilot study was performed at the Department of Hematology, University Medical Center Groningen (UMCG), Groningen, the Netherlands. Patients aged ≥ 18 years undergoing HSCT and receiving anti-infective prophylaxis (ciprofloxacin, fluconazole, or valacyclovir) as routine clinical care were eligible for inclusion. Patients deemed unsuitable to participate at inclusion (e.g., critical illness resulting in severely reduced life expectancy) as judged by the attending physician were not included in the study. Written informed consent was obtained from each patient. The study was evaluated by the Medical Ethics Committee of UMCG and considered to have a negligible risk due to the observational nature of the study (METc 2019/073).

The primary objective was to describe the exposure of the previously mentioned anti-infectives during different stages of mucositis (as defined by citrulline concentrations) and analyze the relationship between anti-infective drug and citrulline concentrations. The secondary outcome was to investigate the composition of the gut microbiota at the baseline level and during mucositis and to explore a potential relationship between gut microbiota and the exposure of anti-infectives.

### Data and sample collection

Available left-over blood samples from routine care were collected daily from the day of transplant up to 14 days after autologous stem cell transplantation. EDTA tubes containing left-over blood were centrifuged at 2000 rcf for 5 min. The resultant plasma was used to measure ciprofloxacin, fluconazole, acyclovir, and citrulline concentrations (drug bioassays described in the supplementary material). The limit of quantification for acyclovir was 0.1 mg/L, for ciprofloxacin was 0.1 mg/L, and for fluconazole was 0.5 mg/L. The bioassays are further described in the supplementary materials. Available left-over fecal swabs (ESwabs, Copan Diagnostics Inc., Brescia, Italy) were collected at weekly intervals from the laboratory of Medical Microbiology and Infection Control of the UMCG in the same time period. The fecal swabs were used to characterize the composition of the gut microbiota.

The following data was collected from electronic patient files: demographic data (sex, age, weight, and height), presence of dialysis, presence of gastrointestinal tubes, documented vomiting or diarrhea, comedication, drug regimen for the analyzed anti-infective prophylaxis (dose, frequency, and route of administration), any plasma or serum concentrations of fluconazole, ciprofloxacin, or acyclovir obtained in routine care, C-reactive protein (CRP) values, and routine blood tests. Ciprofloxacin was administered twice daily as 500 mg orally or 400 mg intravenously, valacyclovir 500 mg twice daily orally, and fluconazole 200 mg once daily either orally or intravenously. Grouping of samples was performed according to previous studies that show severe mucositis symptomology after day 4 after HSCT [9, 13, 19]. As such, samples were divided according to the time of sampling with “mucositis phase I” corresponding to day − 10 (before HSCT) to 4, “mucositis phase II” corresponding to day 5–14.

### Mucositis assessment

Plasma citrulline was measured in 30 μL of plasma using automated ion-exchange column chromatography (Waters, Milford, USA) as previously described [11, 14]. The precision and accuracy were reported as interday CV% of < 3.9% and recoveries ranged from 98.0 to 100%. Levels below 10 μmol/L indicated hypocitrullinemia and considered to represent severe gastrointestinal mucositis. The limit of quantification was 0.3 μmol/L with 10 μL sample.

### Microbiome analysis; 16S rRNA gene sequencing

The fecal microbiota composition was assessed using 16S rRNA gene sequencing as previously described, with few modifications [20–22]. The analysis is further described in the supplementary materials.

### Statistical analysis

Numerical variables were presented with medians and interquartile range (IQR), categorical variables by frequencies and percentages. Spearman’s correlation test was performed between all concentrations obtained on oral anti-infective therapy and citrulline values. The difference between citrulline and different mucositis phases and drug concentrations (obtained on oral therapy) and different mucositis phases were done with the Wilcoxon test, which was corrected for multiple testing. The criteria to include the drugs in the analysis were above limit of quantification, reached steady state and measurement on oral therapy.

A $p$ value of < 0.05 was considered statistically significant and correction for multiple hypothesis testing used, except in the case of small test numbers, in which case the Benjamini-Hochberg (BH) correction was used. Statistical analysis and graphs were performed with R version 3.3.3 and 4.0.5.

## Results

### Patient characteristics

From September 2019 to January 2021 including a temporary recruitment stop due to the COVID-19 pandemic, 21 patients with a median age of 58 (IQR 54–64) years were included in the study. At inclusion, no patients were deemed unsuitable to participate. Eleven patients (52%) had multiple myeloma, and high-dose melphalan was the main (57%) conditioning regimen used. None of the patients were excluded due to the exclusion criteria. The majority of the patients ($n=18$) received concomitant benzylpenicillin, 8 patients received also ceftazidime, single patients received other intravenous anti-infectives (e.g., piperacillin-tazobactam, cefazolin, and caspofungin). The patient characteristics are presented in Table 1. The number of biospecimens ranged from 2 to 20 per patient from day − 10 to 14 with sampling time points differing from each patient. From the left-over samples ($n=252$), 252 citrulline, 110 ciprofloxacin, 80 fluconazole, and 68 acyclovir trough concentrations were obtained on oral therapy. Overall, 48 fecal swabs from 14 patients were analyzed.

**Table 1:** Patient characteristics

Characteristic	Patients ($n=21$)
Age (median, IQR)	58 (54–64)
Male/female ($n$, %)	17/7 (81%/19%)
Weight (kg, median, IQR)	96 (82–105)
Height (cm, median, IQR)	180 (176–188)
Body mass index (kg/m^2^, median, IQR)	27.8 (25.6–29.4)
Underlying disease	
Multiple myeloma Diffuse large B-cell lymphoma Non-Hodgkin’s lymphoma Acute myelogenous leukemia (AML) POEMS syndrome*	11 (52%) 4 (19%) 3 (14%) 2 (10%) 1 (5%)
Conditioning regimen	
Melphalan BEAM (carmustine, cytarabine, etoposide, melphalan) Cyclophosphamide, busulfan	12 (57%) 7 (33%) 2 (10%)
Intravenous anti-infective use	
Benzylpenicillin Ceftazidime Vancomycin Phenoxymethylpenicillin Piperacillin-tazobactam Tobramycin Cefazolin Caspofungin Voriconazole	18 (86%) 8 (38%) 4 (19%) 1 (5%) 1 (5%) 1 (5%) 1 (5%) 1 (5%) 1 (5%)
Anti-infective prophylaxis	
- Ciprofloxacin - Fluconazole - (Val)acyclovir	19 (91%) 17 (81%) 11 (52%)
Plasma measurements ($n=252$)	
Citrulline (number of patients) Median (IQR) per patient	252 (21) 13 (10–13)
Ciprofloxacin (number of patients) Median (IQR) per patient	110 (19) 5 (3–7)
Fluconazole (number of patients) Median (IQR) per patient	80 (13) 3 (3–9)
Acyclovir (number of patients) Median (IQR) per patient	68 (10) 4 (3–12)
Fecal samples (number of patients) Median (IQR) per patient	48 (14) 3 (2–4)

^*^POEMS syndrome = polyradiculoneuropathy, organomegaly, endocrinopathy, monoclonal plasma cell disorder, skin changes

IQR, interquartile range

### Citrulline and anti-infective drug concentrations in plasma

**Fig. 1:**
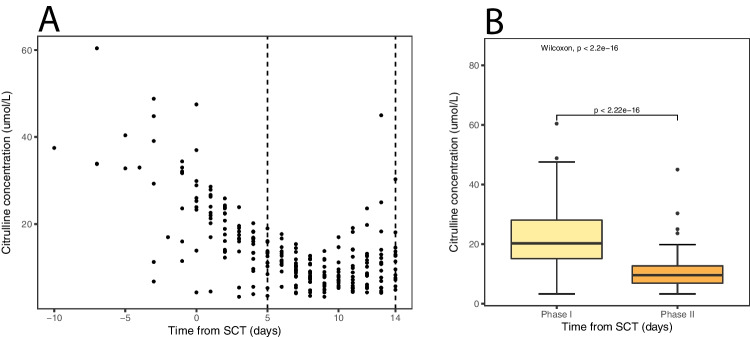
Citrulline concentrations over time for HSCT patients. **A** All measured citrulline concentrations over time from HSCT; **B** citrulline concentrations during two stages of gastrointestinal mucositis (phase I: day − 10 to 4 and phase II: day 5–14)

All included patients suffered from gastrointestinal mucositis as indicated by plasma citrulline concentrations. The median citrulline concentration was 20.2 (IQR 15.1–28.1) μmol/L at mucositis phase I and 9.6 (IQR 6.9–12.7) μmol/L for mucositis phase II. In all patients, a dynamic decrease in plasma citrulline levels was observed over time (Fig. 1A). Subgroup analysis showed a significant decrease in plasma citrulline concentration from mucositis phase I to mucositis phase II (Fig. 1B).

For ciprofloxacin, the median *C*_min_ was 0.3 (range 0.1–1.6) mg/L. 66% (45 samples) of acyclovir trough concentrations were below the limit of quantification, and thus, the median was 0.1 (range 0.1–0.3) mg/L. Three patients (23%) on oral fluconazole therapy reached steady state. The overall median was 5.5 (range 0.3–14.8) mg/L.

**Fig. 2:**
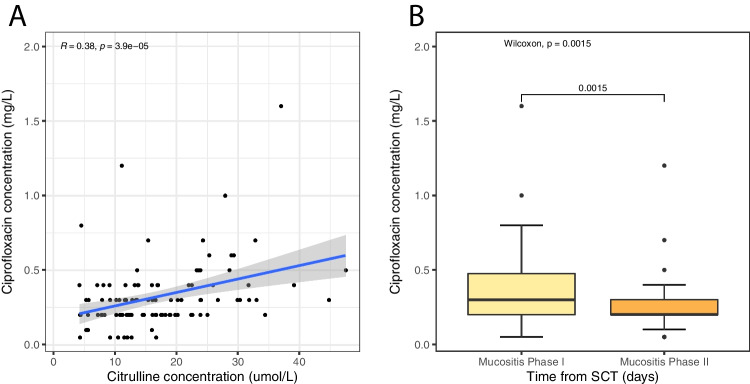
Ciprofloxacin concentrations. The $y$-axis presents the ciprofloxacin concentration (mg/L) and the $x$-axis presents citrulline concentrations (μmol/L) in panel **A** and in panel **B** time from stem cell transplantation (phase I: day − 10 to 4 and phase II: day 5–14)

The correlation analysis was done only for ciprofloxacin as fluconazole concentrations did not reach steady state and acyclovir was measured largely under the limit of quantification. There was a significant correlation observed between ciprofloxacin and citrulline concentrations ($R = 0.38$, *p* = 3.9*10^−5^, Fig. 2A), and a significant difference of ciprofloxacin concentrations was observed between phase I and phase II (Wilcoxon’s $p = 0.0015$, Fig. 2B).

### The influence of the gut microbiota on drug exposure

Analysis of fecal swabs was not possible for all patients, due to insufficient fecal material. Therefore, only 14 patients were included in the analysis of the gut microbiota (median of 3 samples per patient). In this cohort, contrarily to conditioning regimens with BEAM and melphalan that included a considerable number of patients ($n=4$ and $n=9$, respectively), conditioning regimen with cyclophosphamide and busulfan only included 1 patient. This patient was excluded from the analysis as there was not enough material to analyze trends. For this analysis, only the ciprofloxacin levels were included as these were available for the time points of the fecal swabs (Table S1).

**Fig. 3:**
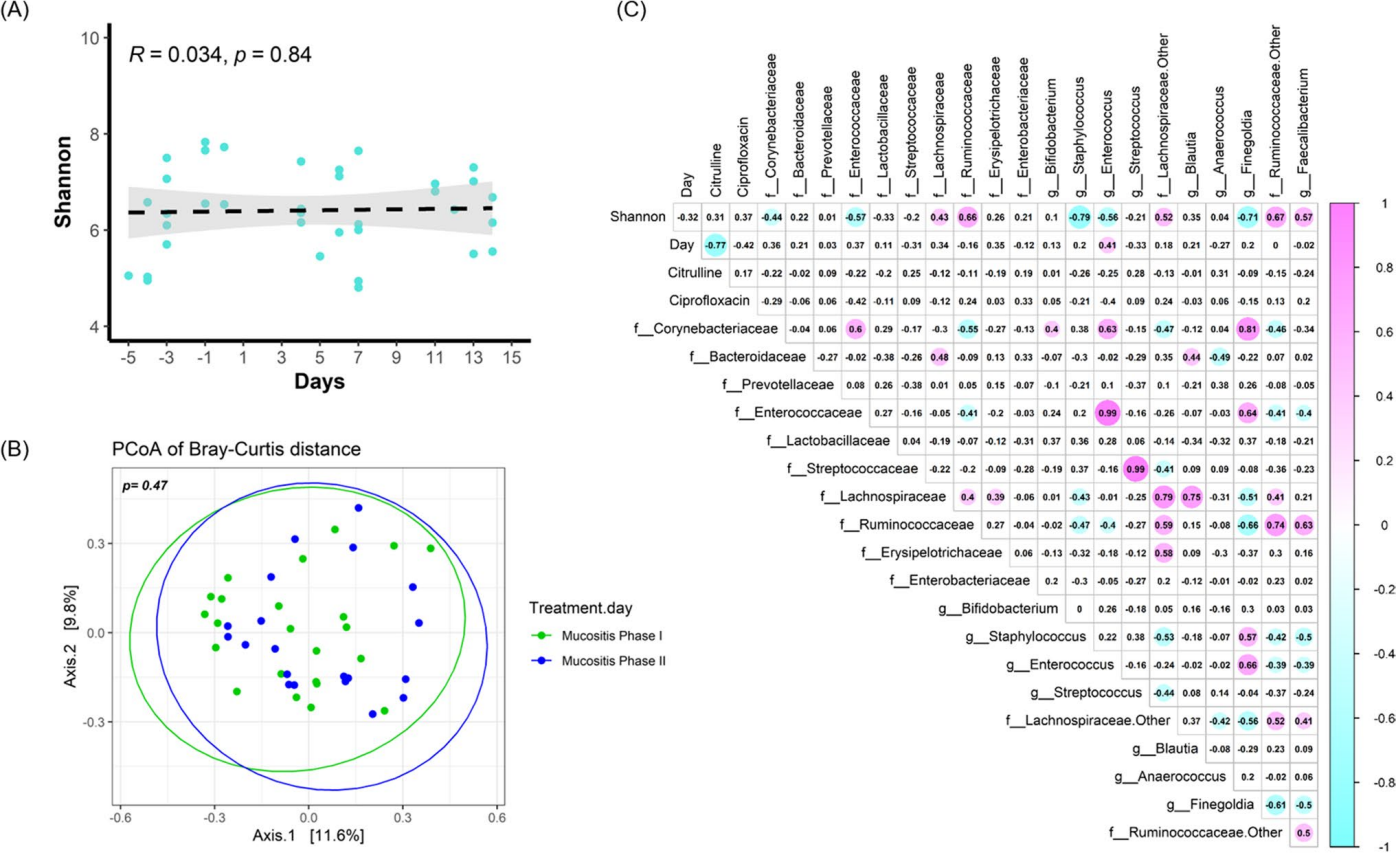
Dynamics of microbial diversity over time and conditioning regimen and Pearson correlation analysis between gut microbiota and plasma citrulline and anti-infective drugs. **A** No differences in microbial diversity were observed throughout time, as indicated by Shannon. **B** Linear regression shows a strong correlation between citrulline levels and day (*R*.^2^ = 0.62, $p<0.001$). **C** Heatmap of Pearson’s correlation coefficient matrix ($R$ values are labeled and BH-corrected $p$ values ($p < 0.05$) are indicated with color background)

Microbial diversity, as represented by Shannon index, throughout showed no significant differences over time (Fig. 3A). Similarly, the ordination plot based on beta diversity (Bray–Curtis distance on the taxonomic level of ASV) showed no separation of samples by mucositis phase ($p=0.47$; ADONIS) (Fig. 3B). The relationship between ciprofloxacin exposure and individual genera/family is presented in Fig. 3C.

## Discussion

This pilot study attempted to describe the exposure of anti-infectives during different stages of gastrointestinal mucositis, defined by plasma citrulline concentrations. We showed significant association between ciprofloxacin concentrations and plasma citrulline concentration. Interestingly, in the 16S rRNA analysis, there was no significant alterations in microbial diversity over time and ciprofloxacin exposure did not impact any specific individual genera/family.

The analysis showed a significant but a weak correlation between ciprofloxacin and citrulline concentrations. The potential lack of a strong correlation between the parameters could be explained by the limitations in sample frequency for each drug and clinical parameter per patient. To truly assess the absorption and exposure differences of these specific anti-infectives, multiple time points per day are needed, e.g., a pharmacokinetic curve containing 3 samples based on a limited sampling strategy. In addition, the timeframe of this study was relatively short; thus, the patients did not have full recovery of mucositis during the 14 days of data collection.

Recently, the gut microbiota has received great attention due to its influence on gastrointestinal mucositis pathobiology [23–25]. In fact, evidence now supports the role of host-microbe interactions in the development of mucositis, with changes in its composition coinciding with the development of mucositis symptomology [5, 26]. Additionally, the gut microbiota has been increasingly recognized for its influence on the toxicity profile of anticancer agents [26]. In fact, the “TIMER” model recently proposed by Alexander et al. eloquently describes the involvement of bacteria in Translocation, Immunomodulation, Metabolism, Enzymatic degradation, and Reduced diversity, all of which have a crucial impact on treatment efficacy and toxicity [26]. As such, we investigated the role of the gut microbiome in this cohort and explored associations between bacterial composition and drug exposure as there is emerging data showing that the gut microbiota may influence the exposure of drug. However, no significant associations were observed between individual genera/family and ciprofloxacin, which may suggest that this antibiotic did not influence the gut microbiota composition, at least within the timeframe in which the samples were collected. In addition, the absence of microbiota changes could also be explained due to the absence of wide spectrum anti-infective use in the population. This result could be explained by bacteria being able to store drugs intracellularly without chemically modifying them as recently suggested by Klünemann et al. [27]. It is however important to acknowledge that the limited number of samples per patient could also explain the lack of significant alterations in the gut microbiota composition observed and therefore our interpretation on the impact of ciprofloxacin on the gut microbiota. Moreover, within the patient group, severe blood stream infections were not documented; however, the absence of these can be assumed due to the limited use of wide spectrum anti-infectives. The anti-infectives used for blood stream infections have an impact on the microbiome; thus, more severe damage might not have been present in our study population.

In this pilot study, we observed a weak correlation between ciprofloxacin and citrulline concentrations, which could suggest that underexposure of ciprofloxacin can occur during severe mucositis. A follow-up study using frequent sampling rather than the use of left-over would be required to investigate the relationship between gastrointestinal mucositis, drug exposure, and gut microbiome.

## Supplementary Information

Below is the link to the electronic supplementary material.

Supplementary file1 (DOCX 25 KB)

## Data Availability

The custom code is available on request from the principal investigator.
