# Supplementary material for: Exposure of anti-infective drugs and the dynamic changes of the gut microbiota during gastrointestinal mucositis in autologous stem cell transplant patients: a pilot study
Source: Ann Hematol. 2023 Jan 17;102(2):421–7. doi: 10.1007/s00277-023-05091-y (PMC9844184; doi:10.1007/s00277-023-05091-y)
Supplement: Supplementary file 1 — Supplementary file1 (DOCX 25 KB) [file 277_2023_5091_MOESM1_ESM.docx]

**Supplementary material**

**Bioanalysis**

Fluconazole, ciprofloxacin, and acyclovir concentrations were measured using a validated liquid chromatography-tandem mass spectrometry assay (Thermo Fisher Scientific triple quadrupole Quantiva MS/MS system with a Thermo Fisher Scientific Vanquish UPLC system, Waltham, MA, USA). The methods were validated in accordance with the Guidance for Industry Bioanalytical Method Validation of the Food and Drug Administration (FDA) and Guideline on bioanalytical method validation of the European Medicines Agency (EMA)[20, 21].

***Fluconazole***

For the sample preparation 500 μL precipitation reagent (methanol containing 0.1 mg/L fluconazole-^2^H_4_) was added to 10 μL plasma. The sample was vortexed for 1 min and centrifuged for 5 min at 9500 rcf. After this, 0.5 μL of the supernatant was injected into the LC-MS/MS system. For fluconazole the linear range was 0.5-50 mg/L with an r^2^ of 0.9975. The validation showed an overall bias ranging from -0.6 % to -5.8 %, a within-run coefficient of variation (CV) ranging from 1.6 % to 4.5 % and a between-run CV% ranging from 0.4 % to 3.6 %.

***Ciprofloxacin***

For the sample preparation 500 μL precipitation reagent (methanol containing 0.2 mg/L ciprofloxacin-^2^H_8_) was added to 100 μL plasma. The sample was vortexed for 1 min and centrifuged for 5 min at 9500 rcf. After this, 0.5 μL of the supernatant was injected into the LC-MS/MS system. For ciprofloxacin the linear range was, 0.1-10 mg/L with an r^2^ of 0.9982. The validation showed an overall bias ranging from 6.1 % to -9.5 %, a within-run CV% ranging from 2.2 % to 6.1 % and a between-run CV ranging from 0.0 % to 2.1 %.

***Acyclovir***

The analysis based on a previously reported bioassay[22]. For the sample preparation 500 μL precipitation reagent (methanol containing 0.5 mg/L acyclovir-^2^H_5_) was added to 100 μL plasma. The sample was vortexed for 1 min and centrifuged for 5 min at 9500 rcf. After this, 0.2 μL of the supernatant was injected into the LC-MS/MS system. For acyclovir the analytical range was, 0.1-20 mg/L with an r^2^=0.9991. The validation showed an overall bias ranging from -1.7 % to 5.3 %, a within-run CV% ranging from 0.9 % to 2.8 % and a between-run CV ranging from 1.4 % to 7.7 %.

For all measurements if the concentration was under the limit of quantification (LOQ), then half of the LOQ was recorded.

**Microbiome analysis; 16S rRNA Gene Sequencing**

To optimize DNA isolation, ESwab tips were vortexed with lysis buffer (500 mM NaCl, 50 mM Tris-HCl (pH 8), 50 mM EDTA, 4 % SDS) and 3mm glass beads for 5 minutes. The resultant mixture was transferred into a screw-cap tube and DNA was extracted using the double bead-beater procedure and the QIAamp DNA Stool Minikit guidelines [25]. Isolated DNA was quantified using the NanoDrop UV Visible Light Spectrophotometer (Thermo Fischer Scientific) and the V3-V4 region amplified using polymerase chain reaction (PCR)[26]. Each PCR reaction contained 1 μl of 10 μM 341f forward primer, 25 μl Phire HS II Master Mix, 22 μl DNase free water, 1 μl of 10 μM 806r barcoded reverse primer and 1 μl DNA template (100 ng/μl). The sequence of the primers used in this study is listed in Supplementary material 1. PCR products denatured at 98°C for 30 sec and amplified over 31 cycles of 98°C for 5 sec, 50°C for 5 sec, 72°C for 10 sec. Samples were held at 72 °C for 1 min and kept at 4°C until collection. Amplification was confirmed using gel electrophoresis.

Size selection and fragment removal was performed using AMPure XP beads (Beckman Coulter Diagnostics, Brea, California) as per manufacturer’s guidelines, before the final PCR products were normalized to 2mM and pooled to form a single library which was stored at -4^o^C until sequencing. Sequencing was performed using the MiSeq Benchtop Next Generation Sequencer (Illumina, San Diego, California). The paired-end sequencing data received from Illumina software were processed by the software QIIME (version 1.9.11)^33^. Readouts with a quality score below 20 were discarded by QIIME and trimmed by Cutadapt (version 3.3) [29] to increase the quality of the sequence readouts. Denoising (removing chimeric sequences, removing singletons, and dereplication) was done with usearch (version 11.0.667) [30] and vsearch (version 2.15.0) [31]. Taxonomy assignment of representative amplicon sequence variants (ASVs) was performed with QIIME against RDP (Ribosomal Database Project) [32] training set 18 with RDP’s classifier.

Data of 16s rRNA sequencing was normalized using cumulative sum scaling (CSS) normalization for multivariate analysis. The calculation of alpha diversity (richness, Chao1, and Shannon) was performed using QIIME. The beta diversity was calculated via Bray-Curtis distances, represented in principal coordinate analysis (PCoA) and performed using R package “phyloseq” (version 1.34.0) and the ADONIS function in the “vegan” package to test significant between groups with 999 permutations. Linear regression and Pearson’s or Spearman’s correlation analysis was performed using R package “base”, “psych” (version 2.1.3) and “corrplot” (version 0.87).

**Table S1.** Clinical data of patients included in the microbial analysis

| **Characteristic** | **Patients (n=14)** |
| --- | --- |
| Faecal samples (number of patients) | 48 (14)  3 (2-4) |
| Conditioning regimen  Melphalan  BEAM (carmustine, cytarabine, etoposide, melphalan)  Cyclophosphamide, busulfan | 9 (64%)  4 (29%)  1 (7%) |
| Anti-infective prophylaxis   - Ciprofloxacin | 11 (78.6%)* |

*No information of ciprofloxacin concentration for all timepoints.
